# Supplementary material for: Smith-specific regulatory T cells halt the progression of lupus nephritis
Source: Nat Commun. 2024 Feb 6;15:899. doi: 10.1038/s41467-024-45056-x (PMC10847119; doi:10.1038/s41467-024-45056-x)
Supplement: Supplementary file 1 — Supplementary Information [file 41467_2024_45056_MOESM1_ESM.pdf]

Smith-specific regulatory T cells halt the progression of lupus nephritis

Peter J Eggenhuizen[1,8], Rachel MY Cheong[1,8], Cecilia Lo[1], Janet Chang[1], Boaz H Ng[1], Yi Tian Ting[1], Julie A Monk[1], Khai L Loh[1], Ashraf Broury[1], Elean SV Tay[1], Chanjuan Shen[2], Yong Zhong[1,3], Steven Lim[4], Jia Xi Chung[1], Rangi Kandane-Rathnayake[1], Rachel Koelmeyer[1], Alberta Hoi[1,5], Ashutosh Chaudhry[6], Paolo Manzanillo[7], Sarah L Snelgrove[1], Eric F Morand[1,5], Joshua D Ooi[1]*

[1]Centre for Inflammatory Diseases, Department of Medicine, School of Clinical Sciences, Monash University, Clayton, Australia.

[2]Dept. of Hematology, The Affiliated Zhuzhou Hospital of Xiangya Medical College, Central South University, Zhuzhou, China.

[3]Dept. of Nephrology, Xiangya Hospital, Central South University, Changsha, China.

[4]Alfred Research Alliance Flow Cytometry Core Facility, Melbourne, Victoria Australia.

[5]Dept. of Rheumatology, Monash Health, Clayton, Victoria, Australia.

[6]Former employee of Amgen

[7]Amgen Research, Amgen Inc, South San Francisco, CA, United States.

[8]Equal contributors

Supplementary Information:

Supplementary Table 1: Characteristics of the top 3 Sm TCRs.

TCR	Variable Gene	CDR3
TCR1	TRAV9-2<br>TRBV11-2	CALSSYGNKLVF<br>CASSSLSGSSYEQYF
TCR2	TRAV29DV5<br>TRBV7-9	CAASRRFGNEKLTF<br>CASRLWSRGRTTEAFF
TCR3	TRAV26-1<br>TRBV7-9	CIVKSWNNRKLIW<br>CASLKRTGGHNQPQHF

Supplementary Table 2: Crystallographic summary data. Data collection and refinement statistics.

	DR15_Sm2
**Wavelength**	
**Resolution range**	45.24 - 3.126 (3.238 - 3.126)
**Space group**	P 43 21 2
**Unit cell**	95.08 95.08 294.2 90 90 90
**Total reflections**	399837 (23226)
**Unique reflections**	24818 (2406)
**Multiplicity**	16.1 (9.7)
**Completeness (%)**	99.6 (97.8)
**Mean I/sigma(I)**	7.77 (1.81)
**Wilson B-factor**	66.29
**R-merge**	0.0664 (0.4451)
**R-pim**	0.099 (0.440)
**CC1/2**	0.989 (0.683)
**CC***	0.997 (0.901)
**Reflections used in refinement**	24739 (2351)
**Reflections used for R-free**	1222 (131)
**R-work**	0.2027 (0.3040)
**R-free**	0.2523 (0.3463)
**CC(work)**	0.945 (0.842)
**CC(free)**	0.920 (0.766)
**Number of non-hydrogen atoms**	6253
**macromolecules**	6224
**ligands**	28
**Protein residues**	762

**RMS(bonds)**	0.004
**RMS(angles)**	0.74
**Ramachandran favored (%)**	93
**Ramachandran allowed (%)**	6.8
**Ramachandran outliers (%)**	0.4
**Rotamer outliers (%)**	2.5
**Clashscore**	15.97
**Average B-factor**	54.98
**macromolecules**	54.84
**ligands**	87.12
**solvent**	35.90

Statistics for the highest-resolution shell are shown in parentheses.

Supplementary Figure 1: Sm Treg stability and bystander suppression

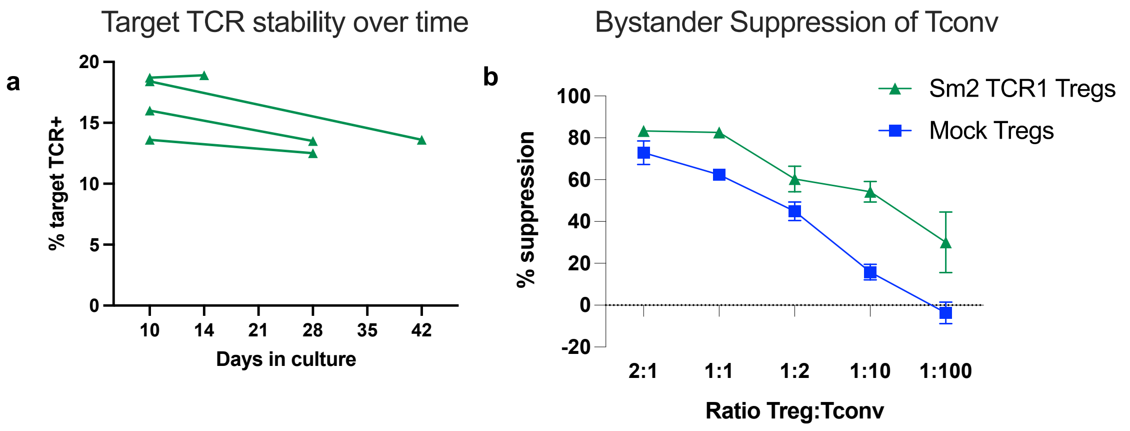

a) Healthy donor Tregs (n=4) transduced with Sm TCR1 maintained the TCR transgene expression over time. Flow cytometry measurements taken between 10 days and 42 days of in vitro expansion culture. b) Bystander suppression of polyclonal Tconv 5-day proliferation in the presence of SmB/B'$_{58\text{-}72}$-pulsed DR15^{+} B-LCLs and titrations of either TCR1-transduced Tregs (green) or polyclonal mock Tregs (blue). % Bystander suppression is measured by the increase in CellTrace Violet (CTV) MFI from polyclonal Tconvs from the baseline proliferation with no Tregs. (n = 2 biologically independent samples). Source data are provided as a Source Data file.

Supplementary Figure 2: Suppressive effect of Sm Tregs on B cells.

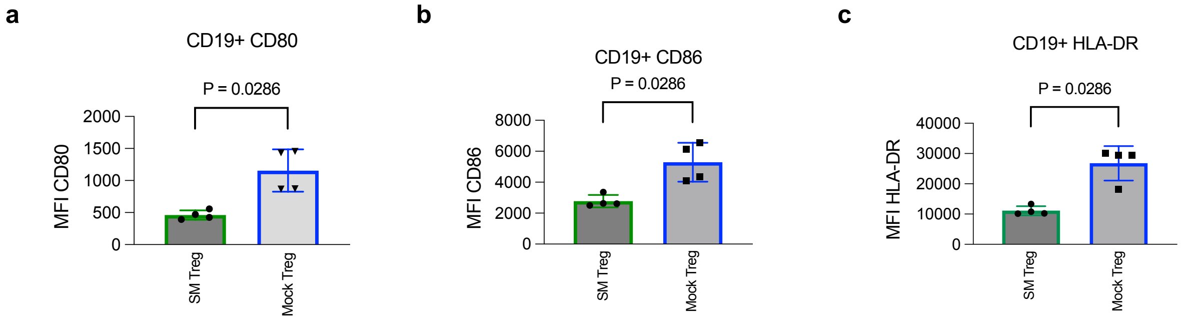

B lymphocytoblastoid (B-LCL) cells from a DR15^{+} healthy donor pulsed with SmB/B'$_{58\text{-}72}$ peptide and cultured for 36hr with either Sm Tregs (green) or polyclonal mock Tregs (blue) show enhanced tolerization by a decrease in expression of **a,** CD80, **b,** CD86 and **c,** HLA-DR on CD19^{+} cells when co-cultured with Sm Tregs compared to polyclonal mock Tregs. P values determined by two-tailed Mann-Whitney test. Source data are provided as a Source Data file.

Supplementary Figure 3: In vitro activation of Sm+ lupus donor Sm Tregs.

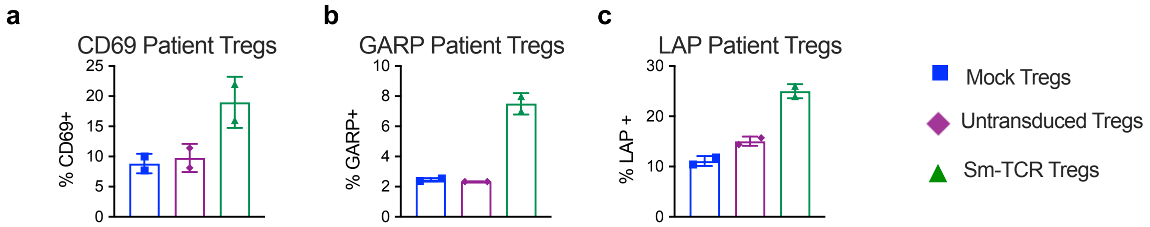

Patient Sm-TCR1 Tregs respond to antigen stimulation better than polyclonal Tregs. Treg activation of patient-derived Tregs measured by the percentage of Tregs expressing Treg-specific activation markers (**b**) GARP and (**c**) LAP and the pan T cell activation marker (**a**) CD69. 10^5 Tregs were stimulated in culture for 5 days with B-LCLs pulsed with SmB/B'$_{58\text{-}72}$ and 10^5 CD4+ Tconv cells in duplicate. Activation was measured by flow cytometry of Tregs transduced with Sm-TCR1 (green) or mock/untransduced (blue/purple). Source data are provided as a Source Data file.

Supplementary Figure 4: In vivo glomerular infiltration of Sm+ lupus donor immune cells.

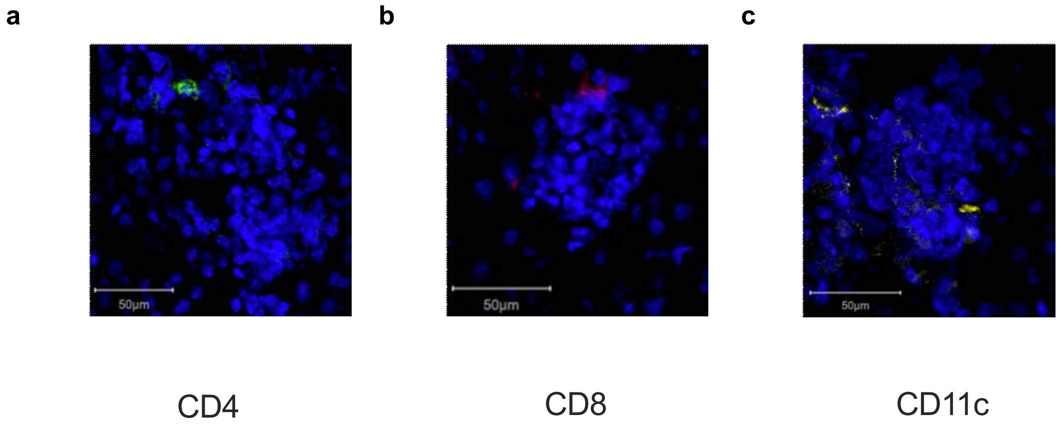

Illustrative immunofluorescent immunohistochemistry showing glomerular infiltration of patient-derived (**a**) CD4+ T cells (green), (**b**) CD8+ T cells (red), and (**c**) CD11c$^+$ dendritic cells (DCs) (yellow) from kidney sections of NSG-MHC^{null} mice at week 8 of the humanised model of lupus nephritis. DAPI staining of nuclei (blue). Experiment was repeated 5 times with similar results.

Supplementary Figure 5: Imaging flow cytometry gating and mask of the immune synapse (IS).

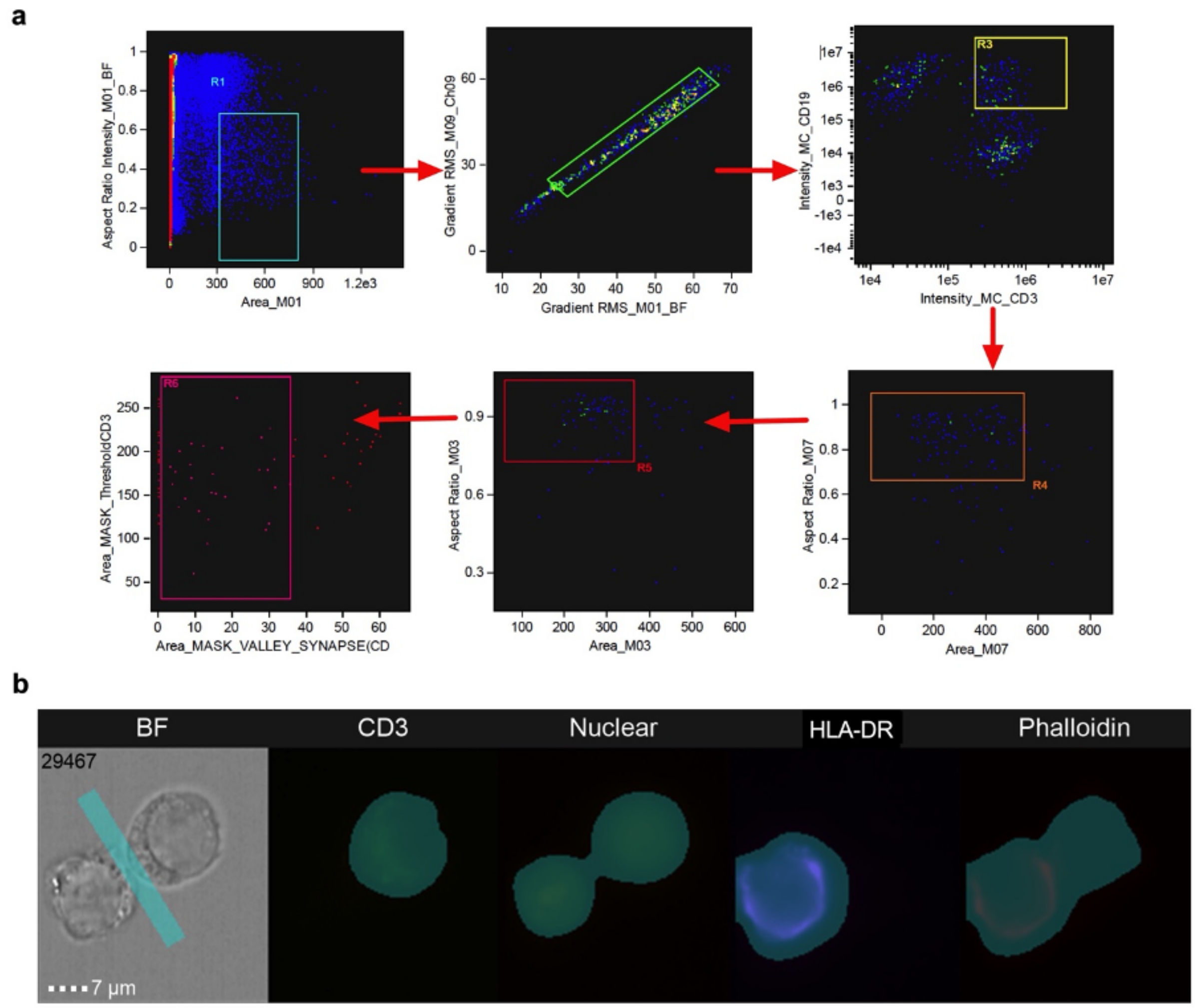

a, Imaging flow cytometry gating strategy used to generate in focus doublets of TCR-transduced J76 Jurkats (CD3) and SmB/B'$_{58\text{-}72}$-pulsed DR15^{+} B-LCLs (HLA-DR). Cells were gated by first selecting the doublet cells by the Area_M01 feature vs. Aspect ratio intensity_M01 feature, followed by gating of in-focus cells by Gradient RMS feature of both brightfield (BF) channels 01 and 09. Doublets double positive for CD3 (Jurkat) and HLA-DR (B-LCL) were then selected based on the intensity_MC feature of CD3 PE (Ch3) vs. HLA DR BV711 (Ch7) followed by further selection of correctly aligned doublets forming immune synapses by Area_M03 vs Aspect ratio_M03 then Area_Mask_Valley_Synapse CD3 vs. Area_Mask_Threshold CD3. **b,** Example of masks (blue shading) of the IS in brightfield (BF) and other masks of Jurkats (CD3), nuclear stain with PI, B-LCLs (HLA-DR) and F-actin cytoskeleton (phalloidin). The mean pixel intensity data of CD3 and F-actin (phalloidin) at the IS were used to quantify the maturity of the immune synapse.

Supplementary Figure 6: Flow cytometry gating strategy for Sm Treg phenotype

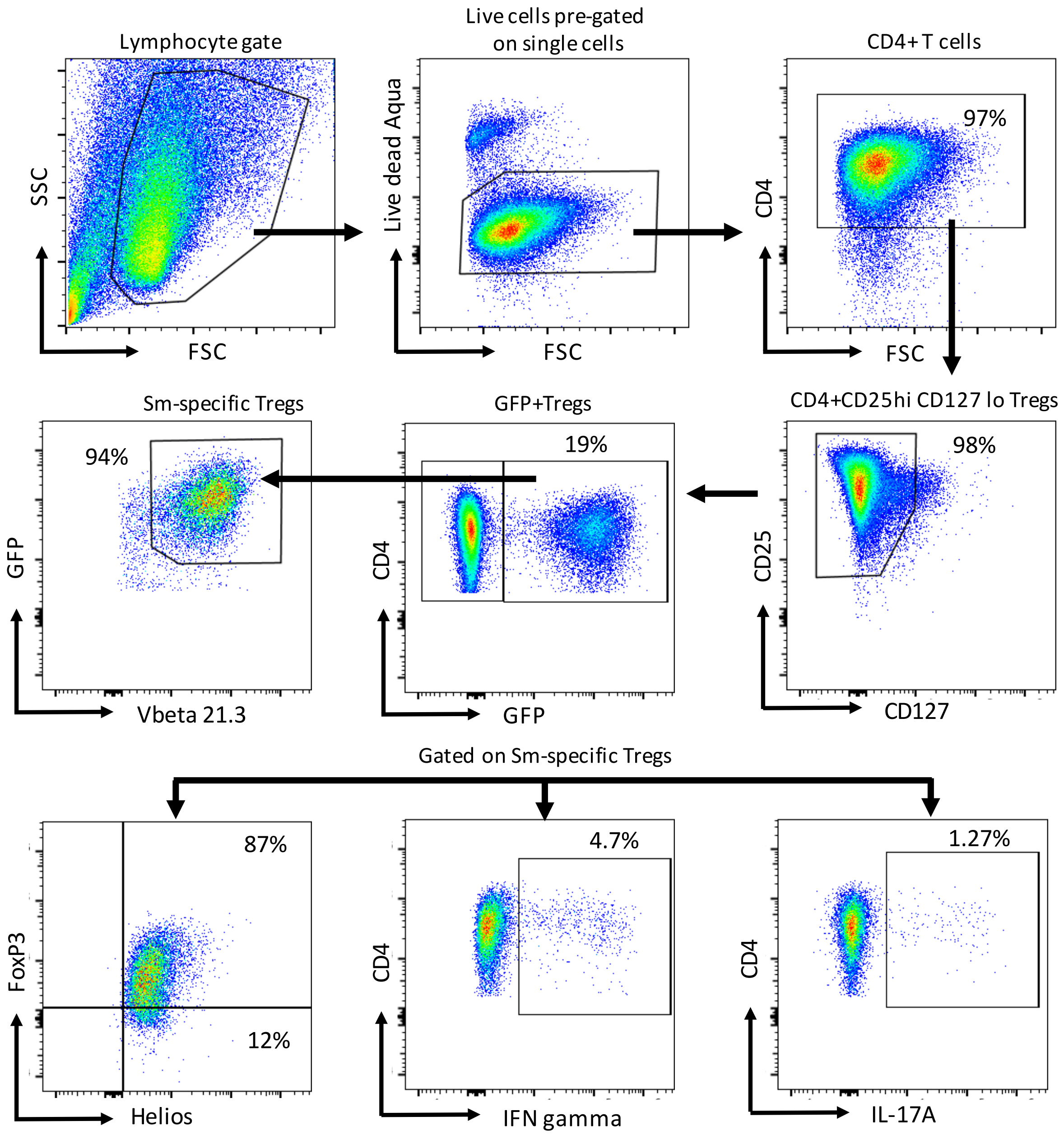

Gating strategy for assessing phenotype of in vitro expanded Sm Tregs. Lymphocytes were gated based on FSC and SSC followed by single cell gate then live cell gating based on Live/dead Aqua negative cells. T cells were then selected based on $CD4^+$ expression. Tregs were then selected based on CD25hi CD127low expression. The transduced GFP^+ Tregs were then selected based on $CD4^+GFP^+$ expression. Sm-specific Tregs were then selected based on GFP^+ TCR Vbeta21.3$^+$ co-expression. If assessing intra-nuclear expression, Sm-specific Tregs were used to quadrant gate FoxP3 and Helios. If assessing intracellular cytokine expression, Sm-specific Tregs were used to gate either $CD4^+$ IFNgamma$^+$ or $CD4^+$ IL-17A$^+$ cells.

Supplementary Figure 7: Flow cytometry gating strategy for Sm Treg suppression assay

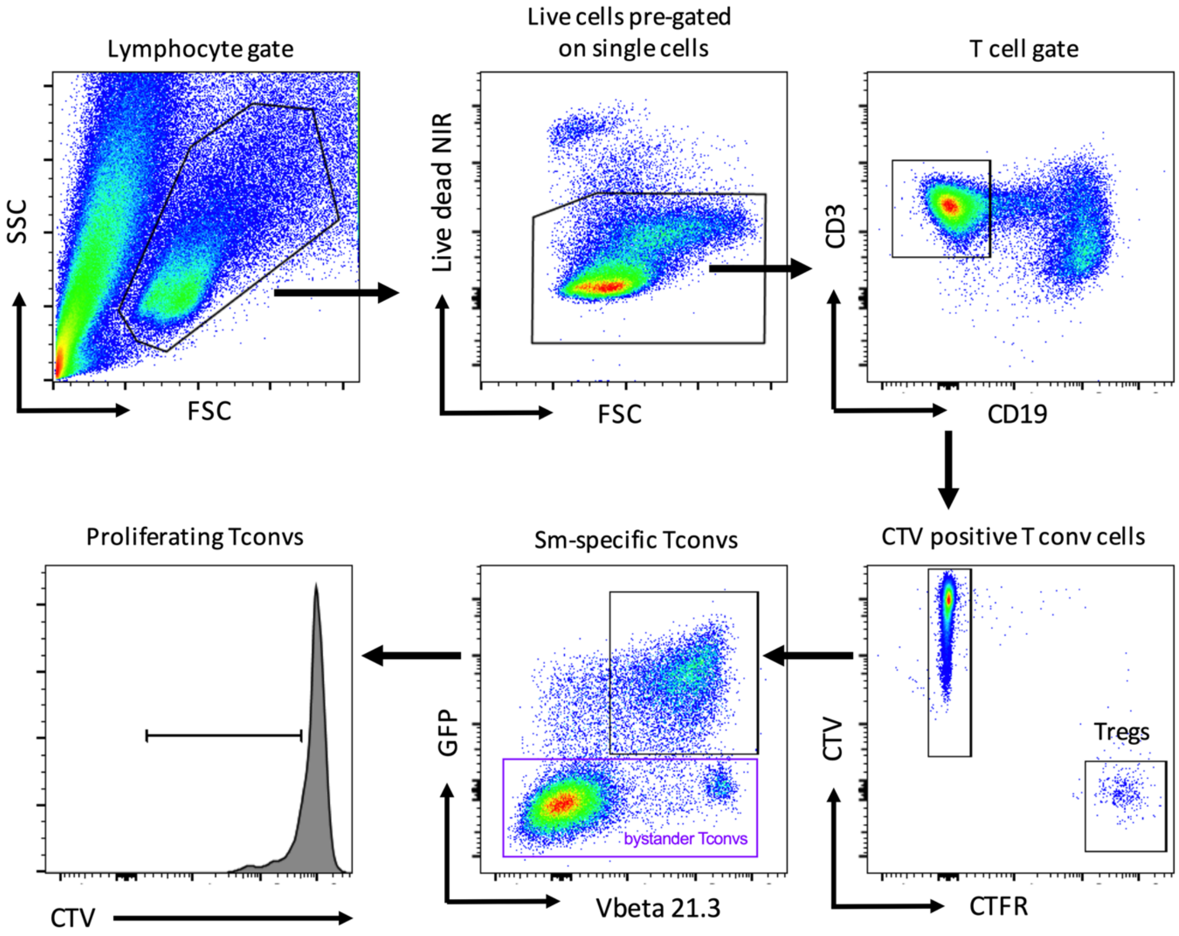

Suppression assay to assess Treg suppressive capacity of Tconvs was analyzed by gating lymphocytes based on FSC and SSC followed by single cell gating then live cell gating based on Live/dead Near Infra-Red negative cells. T cells (Tregs/Tconvs) were then selected based on $CD3^{+}$ $CD19^{-}$ expression. Tregs were then selected based on CellTrace Far Red (CTFR) positive staining. Tconvs were selected based on CellTrace Violet (CTV) positive staining. The transduced Sm-specific Tconvs were then selected based on GFP^{+} TCR Vbeta21.3^{+} expression and the CTV mean fluorescence intensity of this gate used for Sm-specific suppression analysis. The untransduced Sm-specific Tconvs were selected based on GFP- and the CTV mean fluorescence intensity of this gate used for suppression analysis of bystander suppression.

Supplementary Figure 8: Gating strategy for fluorescence activated cell sorting of patient PBMCs, monocytes, and Tregs.

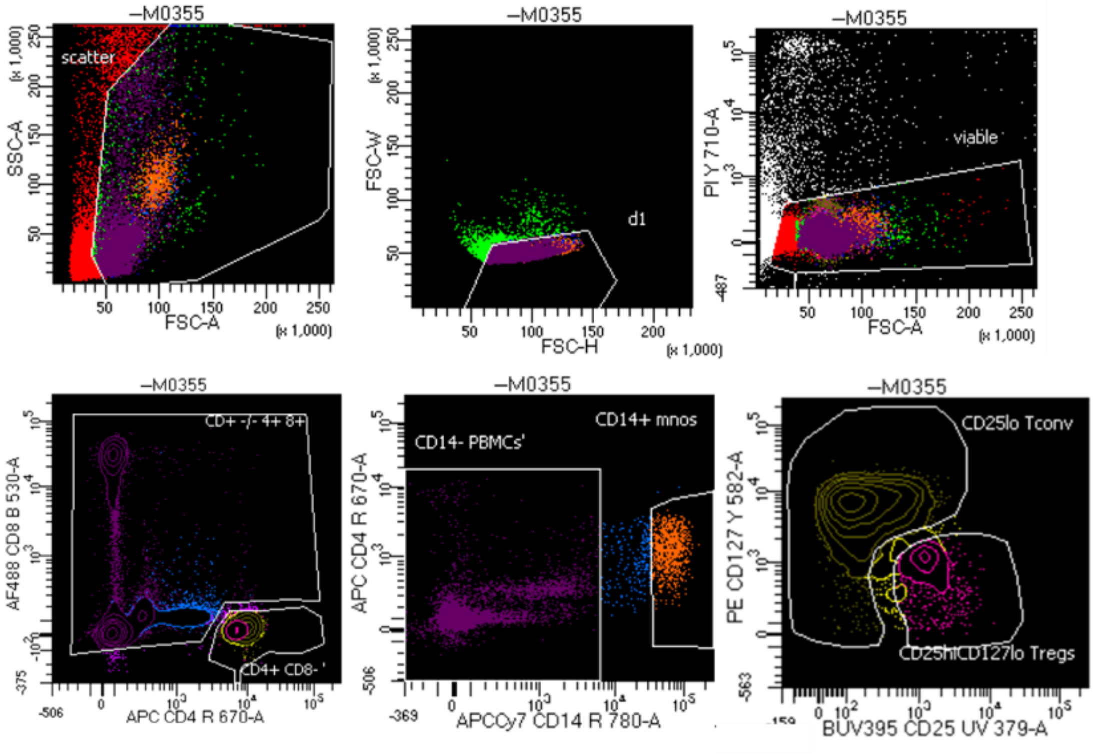

Patient cells were sorted into four groups according to their cell surface markers. These four groups were (1) CD8$^+$CD4$^+$CD14$^-$ PBMCs; (2) CD8$^+$CD4$^+$CD14$^+$ monocytes; (3) CD4$^+$CD8$^-$CD25^{lo}CD127^{hi} Tconvs; and (4) CD4$^+$CD8$^-$CD25^{hi}CD127^{lo} Tregs.
