# Supplementary material for: Smith-specific regulatory T cells halt the progression of lupus nephritis
Source: Nat Commun. 2024 Feb 6;15:899. doi: 10.1038/s41467-024-45056-x (PMC10847119; doi:10.1038/s41467-024-45056-x)
Supplement: Supplementary file 3 — Reporting Summary [file 41467_2024_45056_MOESM3_ESM.pdf]

nature portfolio

Corresponding author(s): Joshua Daniel Ooi

Last updated by author(s): 2023-12-18

Reporting Summary

Nature Portfolio wishes to improve the reproducibility of the work that we publish. This form provides structure for consistency and transparency in reporting. For further information on Nature Portfolio policies, see our Editorial Policies and the Editorial Policy Checklist.

Statistics

For all statistical analyses, confirm that the following items are present in the figure legend, table legend, main text, or Methods section.

n/a	Confirmed	
☐	☒	The exact sample size (*n*) for each experimental group/condition, given as a discrete number and unit of measurement
☐	☒	A statement on whether measurements were taken from distinct samples or whether the same sample was measured repeatedly
☐	☒	The statistical test(s) used AND whether they are one- or two-sided<br>*Only common tests should be described solely by name; describe more complex techniques in the Methods section.*
☒	☐	A description of all covariates tested
☐	☒	A description of any assumptions or corrections, such as tests of normality and adjustment for multiple comparisons
☐	☒	A full description of the statistical parameters including central tendency (e.g. means) or other basic estimates (e.g. regression coefficient) AND variation (e.g. standard deviation) or associated estimates of uncertainty (e.g. confidence intervals)
☐	☒	For null hypothesis testing, the test statistic (e.g. *F*, *t*, *r*) with confidence intervals, effect sizes, degrees of freedom and *P* value noted<br>*Give P values as exact values whenever suitable.*
☒	☐	For Bayesian analysis, information on the choice of priors and Markov chain Monte Carlo settings
☒	☐	For hierarchical and complex designs, identification of the appropriate level for tests and full reporting of outcomes
☒	☐	Estimates of effect sizes (e.g. Cohen's *d*, Pearson's *r*), indicating how they were calculated

Our web collection on statistics for biologists contains articles on many of the points above.

Software and code

Policy information about availability of computer code

Data collection	For flow cytometry acquisition: BD FACSDiva software version 8.0.1 on an LSR-Fortessa X20 flow cytometer (BD) or Spectroflo Software (Cytek, version 3.1.2) on an Aurora flow cytometer (Cytek).<br>For Imaging flow cytometry: Inspire software (Luminex) on an Amnis Imagestream X Mark II (Luminex).<br>For immunofluorescence histology: VS120 slide scanner (Olympus Life Science) using OlyVIA Software (Olympus Life Science)
Data analysis	For scRNA Seq: Cell Ranger (10X Genomics, version 3.0.2), Loupe Cell Browser (10X Genomics, version 4.2.0) and Loupe VDJ Browser (10X Genomics, version 3.0.0).<br>For flow cytometry analysis: FlowJo version 10.9.0 and and GraphPad Prism version 8.3.1.<br>For Imaging flow cytometry analysis: Ideas Software version 6.2 (Luminex) and GraphPad Prism version 8.3.1.<br>For immunofluorescence histology: QuPath (https://qupath.github.io/; University of Edinburgh).

For manuscripts utilizing custom algorithms or software that are central to the research but not yet described in published literature, software must be made available to editors and reviewers. We strongly encourage code deposition in a community repository (e.g. GitHub). See the Nature Portfolio guidelines for submitting code & software for further information.

Data

Policy information about availability of data

All manuscripts must include a data availability statement. This statement should provide the following information, where applicable:

- Accession codes, unique identifiers, or web links for publicly available datasets
- A description of any restrictions on data availability
- For clinical datasets or third party data, please ensure that the statement adheres to our policy

Source data are provided with this paper. ScRNASeq data is uploaded to Gene Expression Omnibus accession number GSE242152. Crystallographic data was uploaded to Worldwide Protein Data Bank accession 8TBP and a crystallographic data summary is found in Supplementary Table 2.

Research involving human participants, their data, or biological material

Policy information about studies with human participants or human data. See also policy information about sex, gender (identity/presentation), and sexual orientation and race, ethnicity and racism.

Reporting on sex and gender	Sex and gender was not included in the study design.
Reporting on race, ethnicity, or other socially relevant groupings	Race, ethnicity or other socially relevant grouping were not included in the study.
Population characteristics	Adult SLE patients who fulfilled American College of Rheumatology Classification criteria for SLE and had anti-Sm autoantibodies and positive for HLA-DR15. Covariate population data: sex (0 male, 5 female), age range (28-58 years).
Recruitment	Adult SLE patients who fulfilled American College of Rheumatology Classification criteria for SLE and had anti-Sm autoantibodies and positive for H LA-DR15.
Ethics oversight	Monash Health Human Research Ethics Committees and Monash University.

Note that full information on the approval of the study protocol must also be provided in the manuscript.

Field-specific reporting

Please select the one below that is the best fit for your research. If you are not sure, read the appropriate sections before making your selection.

- [x] Life sciences
- [ ] Behavioural & social sciences
- [ ] Ecological, evolutionary & environmental sciences

For a reference copy of the document with all sections, see nature.com/documents/nr-reporting-summary-flat.pdf

Life sciences study design

All studies must disclose on these points even when the disclosure is negative.

Sample size	Sample size was chosen based on the number of SLE patient donors that were available. Based on the relatively consistent level of disease suppression, the sample size was sufficient to measure a statistically significant result.
Data exclusions	No data was excluded from analysis.
Replication	The experiment was replicated 5 times. Each replication of the experiment is represented by a single dot point on the graphs.
Randomization	Samples and animals were randomly allocated into groups.
Blinding	Urine samples and histological sections were coded and scored by a blinded person.

Reporting for specific materials, systems and methods

We require information from authors about some types of materials, experimental systems and methods used in many studies. Here, indicate whether each material, system or method listed is relevant to your study. If you are not sure if a list item applies to your research, read the appropriate section before selecting a response.

Materials & experimental systems

n/a	Involved in the study	
	x	Antibodies
	x	Eukaryotic cell lines
x		Palaeontology and archaeology
	x	Animals and other organisms
x		Clinical data
x		Dual use research of concern
x		Plants

Methods

n/a	Involved in the study	
x		ChIP-seq
	x	Flow cytometry
x		MRI-based neuroimaging

Antibodies

Antibodies used	anti-human CD4 BUV496 (clone SK3, BD), anti-human CD8 APC-H7 (clone SK1, BD), anti-human CD69 BV711 (clone FN50, BD), anti-human HLA-DR AF488 (clone L243, Biolegend), anti-human HLA-DR BV421 (clone G46-6, BD), anti-human CD3 PE (clone OKT3, Invitrogen), anti-human CD4 BUV496 (clone SK3, BD), anti-human CD127 APC-Vio770 (clone REA614, Miltenyi), anti-human CD25 APC (clone BC96, Biolegend) and anti-human TCR Vbeta21.3 PE (clone REA894, Miltenyi), anti-human Foxp3 (clone 236A/E7, BD), anti-human Helios (clone 22F6, Biolegend), anti-human IFN-gamma (clone B27, BD) and anti-human IL-17A (clone N49-653, BD), anti-human CD4 R718 (clone SK3, BD), anti-human CD69 BUV395 (clone FN50, BD), anti-human GARP BV786 (clone 7B11, BD), anti-human CD3 PerCP (clone SK7, Biolegend), anti-human CD19 PE-CF594 (clone HI519, BD), anti-human CD4 Pacific Blue (clone RPA-T4, Biolegend), anti-human CD8 AF488 (clone HIT8a, Biolegend), anti-human CD127 PE (clone A019D5, Biolegend), anti-human CD25 BUV395 (clone 2A3, BD), anti-human CD14 APC-Cy7 (clone MOP9, BD), anti-human LAP PE-Cy7 (clone FNLAP, Invitrogen), anti-human GARP (clone BB700, BD) and anti-human CD69 BV711 (clone FN50, BD).
Validation	The certificates of analysis found on the supplier websites contain the validation data that these products have been manufactured and tested in accordance with the manufacturer's specifications.

Eukaryotic cell lines

Policy information about cell lines and Sex and Gender in Research

Cell line source(s)	Viral Production Cells (Gibco). J76 Jurkat cells sourced from Mirijam Heemskerk are human T cell line as characterised in the literature. Expi293F (GnTI-/-) cells were sourced as a kind gift from the Whisstock Laboratory, Monash University.
Authentication	Viral production cells (Gibco) were authenticated by the supplier in the Certificate of Analysis. J76 Jurkat cells were authenticated by phenotypic characterisation by flow cytometry.
Mycoplasma contamination	Cell lines were not tested for mycoplasma.
Commonly misidentified lines (See ICLAC register)	No commonly misidentified cell lines were used in the study.

Animals and other research organisms

Policy information about studies involving animals; ARRIVE guidelines recommended for reporting animal research, and Sex and Gender in Research

Laboratory animals	Mus musculus NOD.Cg-PrkdcscidH 2-Ablem1Mvw H2-Kltm1Bpe H2-Dltm1Bpe Il2rgtm1Wjl/SzJ, JacksonL aboratories strain 025216. Mice started experiment at 8-10 weeks old. Mice are housed in Tecniplast IVC caging with the room temperature and humidity maintained between 18 - 24 degrees Celsius and 40 - 70% Relative humidity as per the required Australian Code of Practice for housing of laboratory animals. The average ambient temperature in the room is generally between 20 - 22 degrees and RH is generally 45 - 50%. The room is maintained on a 12 hour automated light/dark cycle whereby the light cycle begins at 6.30am and dark cycle begins at 6.30pm (Australian Eastern Standard Time) or light cycle begins at 7.30am and dark cycle begins at 7.30pm (Australian Eastern Daylight Time).
Wild animals	Study did not involve wild animals.
Reporting on sex	Sex was not considered in the study design. Mice were assigned randomly to groups.
Field-collected samples	Study did not involve field collected samples.
Ethics oversight	Monash Animal Ethics Committee

Note that full information on the approval of the study protocol must also be provided in the manuscript.

Flow Cytometry

Plots

Confirm that:

- [x] The axis labels state the marker and fluorochrome used (e.g. CD4-FITC).
- [x] The axis scales are clearly visible. Include numbers along axes only for bottom left plot of group (a 'group' is an analysis of identical markers).
- [x] All plots are contour plots with outliers or pseudocolor plots.
- [x] A numerical value for number of cells or percentage (with statistics) is provided.

Methodology

Sample preparation	Samples were first stained with Live/Dead Viability dye then surface stained with antibodies. If intracellular/intranuclear staining was performed, cells were fixed then permeabilized and then stained with antibody. Full details of each step are found in the methods.
Instrument	BD FACS Aria for cell sorting, Cytek Aurora for analysis, Amnis Imagestream X Mark II for imaging flow cytometry.
Software	For flow cytometry acquisition: BD FACSDiva software version 8.0.1 on an LSR-Fortessa X20 flow cytometer (BD) or Spectroflo Software (Cytek, version 3.1.2) on an Aurora flow cytometer (Cytek).<br>For Imaging flow cytometry: Inspire software (Luminex) on an Amnis Imagestream X Mark II (Luminex).<br>For flow cytometry analysis: FlowJo version 10.9.0 and and GraphPad Prism version 8.3.1.<br>For Imaging flow cytometry analysis: Ideas Software version 6.2 (Luminex) and GraphPad Prism version 8.3.1.
Cell population abundance	Abundance of post sort fractions are listed in the gating strategy figures found in the supplementary information.
Gating strategy	Gating strategies used are found in the supplementary information.

- [x] Tick this box to confirm that a figure exemplifying the gating strategy is provided in the Supplementary Information.
